# CD86, the double agent: Significance of CD86 expression in B‐cell malignancies

**DOI:** 10.1002/ijc.70028

**Published:** 2025-07-10

**Authors:** Gábor Barna, Gábor Szalóki, Ágnes Márk, Anna Hunyadi, Csilla Kriston

**Affiliations:** ^1^ Department of Pathology and Experimental Cancer Research Semmelweis University Budapest Hungary

**Keywords:** B‐ALL, CD86, CLL, drug resistance, MM

## Abstract

Bypassing the anti‐tumor functions of the immune system is one of the keys to tumor survival. Tumors, especially hematological tumors, produce or express factors that retune T and B cells to inhibit the immune response. Coreceptors CD28 and CTLA‐4, and their ligands, CD80 and CD86, are involved in the immunological synapse and play an important role in immune processes. CD86 is widely expressed in hematological tumors, mainly of B‐cell origin, and only a few studies are available about its role in the pathogenesis. This study discusses the importance of CD86 expression in hematological malignancies. Does its presence help or hinder the development and survival of tumor cells? The answer to this question will facilitate a more profound comprehension of the biology of hematological malignancies and the development of new therapeutic options.

## Graphical abstract

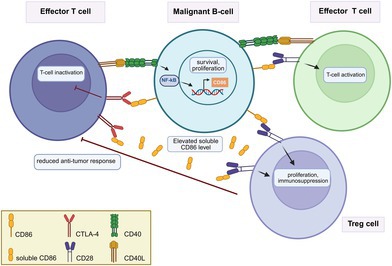

**Abbreviations**
AML: acute myeloid leukemia
APC: antigen‐presenting cell
Ara‐C: cytosine arabinoside
B‐ALL: B‐cell acute lymphoblastic leukemia
BCR: B‐cell receptor
BTK: Bruton's tyrosine kinase
CAR‐T cell: chimeric antigen receptor T cell
CLL: chronic lymphocytic leukemia
CRLF2: cytokine receptor‐like factor 2
CTLA‐4: cytotoxic T‐lymphocyte‐associated protein 4
DC: dendritic cell
FAB: french‐american‐british
IFNγ: interferon‐gamma
IRF4: interferon regulatory factor 4
ITK: IL2‐inducible T‐cell kinase
LN‐CLL: lymph node‐derived CLL cells
LPS: lipopolysaccharide
MAPK: mitogen‐activated protein kinase
MHCII: major histocompatibility complex II
MM: multiple myeloma
NF‐kB: nuclear factor kappa B
PEL: primary effusion lymphoma
Ph‐like: Philadelphia chromosome‐like
PI3K: phosphoinositide 3‐kinase
PKC: protein kinase C
PLCγ2: phospholipase C gamma 2
PMA: phorbol 12‐myristate 13‐acetate
sCD86: soluble isoform of CD86
SMZL: splenic marginal zone lymphoma
TCR: T‐cell receptor
Th: helper T cell
TLR: Toll‐like receptor
Treg: regulatory T‐cell

## 1 INTRODUCTION

Cooperation and communication between T and B cells are key to the functional immune system. This is a tightly regulated process: antigen recognition alone is insufficient for B‐cell activation, but antigen presentation for T cells is also necessary. Several surface ligands (e.g., CD40L) and soluble factor production (e.g., IL‐21) are engaged in the reinforcement of B‐cell activation by T cells.^1^ One of the most important molecules in the immunological synapse is CD86, also known as B7‐2, which plays a role in the activation or possible inhibition of T cells. Several hematological tumors express CD86 on their surface. It is not yet clear whether it helps or hinders the proliferation and survival of tumor cells. In this review, we will detail the presence, structure, and function of this molecule on the surface of various types of cells according to the current literature and our results.

## 2 THE STRUCTURE AND FUNCTION OF CD86

CD86 is a 70‐kDa transmembrane glycoprotein that belongs to the immunoglobulin superfamily. It shows limited structural homology and functional similarity to CD80, also known as B7‐1. The gene encoding CD86 can be found on chromosome 3 (3q13.33) and consists of 7 exons.^2^ In 1993, almost simultaneously, two groups discovered the CD86 (B7‐2) molecule: Azuma M et al. found that there must have been another molecule besides the B7‐1 that binds to CD28 and cytotoxic T‐lymphocyte‐associated protein 4 (CTLA‐4) expressed on T cells. They developed the IT2 antibody that bound to a 70 kDa glycoprotein, which they cloned.^3^ At the same time, Freeman GJ et al. cloned a counter receptor of CD28 and CTLA‐4, which was 26% identical to B7‐1 and was also expressed on non‐stimulated B cells, in contrast to B7‐1, which was expressed only on stimulated B cells.^4^ The screening of different cell types revealed that CD86 is also expressed on other antigen‐presenting cells (APC) like dendritic cells (DCs), monocytes at low levels, and activated T and NK cells.^3^

Regarding their structure, both CD80 and CD86 contain a membrane‐distal Ig V‐type and a membrane‐proximal Ig C‐type domain, of which the V‐type domain plays a role in the CTLA‐4 and CD28 binding and T‐cell activation.^5^ On the intracellular part, CD86 contains a polylysine motif responsible for the cytoskeletal contact. It plays an important role in the cytoskeleton remodeling of APCs and influences immune synapse formation and T‐cell activation.^6^

Later, a soluble isoform of CD86 (sCD86) that lacks the exon 6 encoding the transmembrane region was found in the peripheral blood in healthy people and patients with hematological diseases. In some patients with acute myeloid leukemia (AML) or chronic lymphocytic leukemia (CLL), elevated levels of sCD86 were detected compared to healthy donors. Screening a wide range of normal, non‐malignant cells revealed that sCD86 was only produced by monocytes and DCs.^2^ Stabilized cell lines, some Hodgkin's (e.g., L428, KM‐H2) and B‐cell lymphomas (e.g., Raji, EBV‐1), and a few AML cell lines (e.g., THP‐1, HL60) also produced sCD86. In later studies, sCD86 was also detected in the peripheral blood of patients with multiple myeloma (MM).^7^

## 3 REGULATION OF CD86 EXPRESSION

**FIGURE 1:**
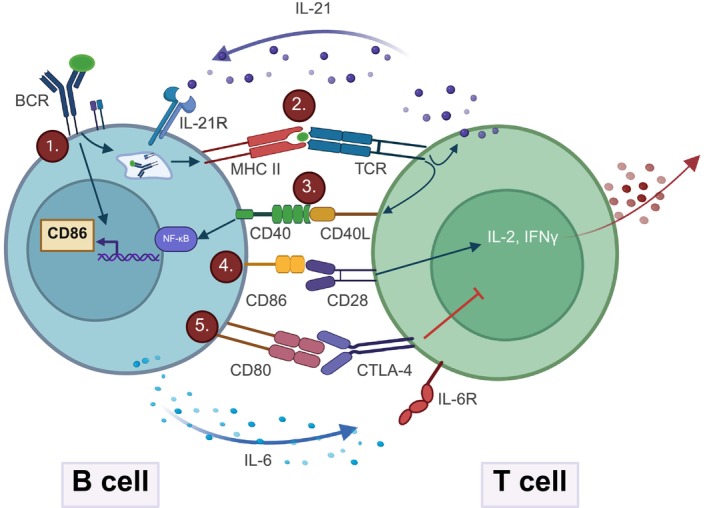
The regulation and function of CD86. The CD86 molecule plays an active role in developing the immunogen or tolerogen response. After the antigen is recognized by BCR (1.) and processed inside the cell, its fragments appear on the surface of the MHCII molecule. During this process, the BCR signaling pathway is activated, and the expression of CD86 is increased. MHCII molecules present the antigen to T cells, which recognize it through their specific T‐cell receptor (2.). This binding induces IL‐21 production and CD40L expression (3.), which further activates B cells via the NF‐kB signaling pathway. NF‐kB activity also increases CD86 expression (4.), which enhances the cytokine production of T cells by binding to CD28. Finally, CD80‐CTLA‐4 binding (5.) inhibits further activation of T cells. BCR, B‐cell receptor; CTLA‐4, cytotoxic T‐lymphocyte‐associated protein 4; IL‐6R/IL‐21R, interleukin‐6/21 receptor; IFNγ, interferon‐gamma; MHCII, major histocompatibility complex II; NF‐kB, nuclear factor‐kB; TCR, T‐cell receptor.

Several factors influence the expression of CD86 on B cells. Early studies revealed that the stimulation of B‐cell receptor (BCR) or lipopolysaccharide (LPS)‐induced B‐cell activation increased the mRNA and surface protein levels of CD86.^8^, ^9^ In addition, IL‐2 and IL‐4 cytokines^10^ and CD40‐CD40L binding also increased CD86 expression.^11^ In a former study, in mice (CBA/N) with mutant Bruton's tyrosine kinase (BTK), the signaling pathway of CD40‐CD40L was investigated. In these mice, both the expression of CD80 and CD86 was inhibited. Ionomycin and Phorbol 12‐myristate 13‐acetate (PMA), a potent activator of protein kinase C (PKC), induced the expression of CD86 but not CD80, indicating that PKC was involved in this process. PKC can activate the MAPK and NF‐kB signaling pathway. Mapping of the promoter region of the CD86 gene revealed an NF‐kB binding site through which expression was enhanced during the B and T cells interaction. Helper T cells (Th) induced CD86 expression on B cells, while regulatory T cells (Treg) inhibited this process by blocking NF‐kB activation (Figure 1).^12^

## 4 WHAT IS THE ROLE OF CD86 IN T‐CELL FUNCTION?

On the surface of T cells, besides the T‐cell receptor (TCR), several costimulatory and coinhibitory receptors, such as CD28 and CTLA‐4 regulate the function of the T cells. In most cases, co‐ligation of CD28 is required for T‐cell activation. This signal helps to stabilize cytokine mRNAs,^13^ activate PI3K and NF‐kB, inducing cytokine production (e.g., IL‐2, IFNγ),^14^, ^15^ and enhanced proliferation. In contrast, CTLA‐4 inhibits T‐cell activation by increasing the motility of T cells, overwriting the TCR‐induced stop signal, and shortening the time of T cell—APC contact, thus reducing cytokine production and T‐cell proliferation.^16^

CD86 binding to CD28 or CTLA‐4 was shown in the first publication.^17^ Subsequent research has investigated the strength and the nature of this contact. Linsey PS et al. showed that CD86 and CD80 bind with low avidity to CD28 and high avidity to CTLA‐4.^18^ Collins et al. discovered from crystal structure analysis that CTLA‐4 formed bivalent bonds while CD28 formed only monovalent bonds.^15^ Different interactions could be found between CD86, CD80, CD28, and CTLA‐4. While CD86 bound more weakly to its receptor in monomeric form, CD80 formed homodimers and bound more strongly. The strongest, highest‐affinity binding was formed between CD80 and CTLA‐4, whereas the weakest was between CD86 and CD28. In addition, CD86 had been shown to bind more effectively to CD28 than CD80.^15^ We have seen that CD80 and CD86 molecules bind differently to their receptors, but why is this important? What kind of process do they set in motion?

The appearances of CD86 and CD28 are similar: CD86 is expressed on the naive, non‐stimulated B cells,^4^ while CD28 is present on the resting conventional T cells as well.^19^ In contrast, CTLA‐4 is not detected on resting T cells and is only observed 2 days after activation. Similarly, CD80 is expressed exclusively by activated B cells.^20^, ^21^ Thus, CD86 first binds to CD28 to stimulate resting T cells, and later, CTLA‐4, expressed by activated T cells, inhibits further activation. The question is which B7 ligand binds to CTLA‐4, as both are expressed on activated B cells and bind more strongly to CTLA‐4 than to CD28. In terms of bond strength, the CD80‐CTLA‐4 is the most likely combination. In this case, however, the CD86‐CD28 binding may persist and result in a certain level of T‐cell activation. It can be seen that this time‐activation‐dependent variation, as well as the different affinity binding, helps to fine‐tune activation inhibition.

These processes could be studied in detail in Treg cells, on which both CD28 and CTLA‐4 are present. CD86 has been found to bind to CD28 and promote Treg proliferation and survival, as well as maintain and induce CTLA‐4, ICOS, and OX40 expression on these cells.^22^

## 5 INTRACELLULAR SIGNALS INDUCED BY CD86

Koorella Ch. et al. investigated CD80/CD86‐induced intracellular signals in dendritic cells (DCs) and found that cross‐linking of CD80/CD86 molecules activated the PI3K/AKT pathway, which induced IL‐6 production via the canonical NF‐kB pathway after Akt phosphorylation.^23^ In other studies, the CD86 signaling pathway was examined in mouse B cells, where CD86 engagement led to the activation of the NF‐κB pathway in activated B cells (Figure 1.). More specifically, this was achieved through the phosphorylation of IκBα, leading to the release and nuclear translocation of NF‐κB dimers and regulating IgG production.^24^, ^25^

## 6 EXPRESSION OF CD86 IN B‐CELL MALIGNANCIES

In the previous sections, we saw that CD86 plays an important role in regulating immune activation and tolerance, specifically the activation of T cells. T cells are the key factors in immune surveillance since they can recognize immunogenic tumor cells through their TCR and destroy them via their cytotoxic function. This mechanism is exploited by CAR‐T cells and bispecific antibody therapies. However, in many tumors, these therapies are ineffective. Let's see whether CD86 may contribute to the loss of cytotoxic function.

### 6.1 Chronic lymphocytic leukemia

CLL is one of the most common lymphoid leukemias in the Western World^26^; therefore, it has been the subject of intensive research from the beginning. Early studies revealed that the expression of two costimulatory molecules (CD80, CD86) is significantly lower on CLL cells than on normal healthy B cells.^27^ This observation is not surprising in the case of CD80 because it is not expressed on non‐stimulated normal B cells, but in the case of CD86, it is unexpected. However, IFNγ treatment elevated the CD86 RNA and protein levels in CLL cells.^27^ Subsequent studies compared the expression of cell surface markers in CLL cells, where the proliferating, Ki67‐positive cells had significantly higher CD86 expression.^28^ Investigating lymphocytes from different zones of healthy lymph nodes showed that light zone cells (centrocytes) were CD86^hi^CXCR4^lo^, while dark zone cells (centroblasts) were CD86^lo^CXCR4^hi^.^29^ CD86^hi^CXCR4^lo^ CLL cells are also present in the peripheral blood. Cell cycle analysis showed that these cells were mainly in the G2/M phase.^30^ In these CD86‐positive cells, the number of γ‐H2AX foci, indicating double‐strand breaks and the activated phenotype, was higher than in the CD86‐negative cells. Furthermore, CD86 expression of CLL cells positively correlated with the RAI stage. The CD86^hi^CXCR4^lo^ population partially overlapped with the CD5^hi^CXCR4^lo^ population, which characterized CLL cells that divided and recirculated into the lymph nodes.^31^ Subsequent studies showed that lymph node‐derived CLL cells (LN‐CLL) exhibited an activated phenotype and higher CD86 expression. These cells could form immune synapses with CD4‐positive T cells. Furthermore, their migration capability was higher than other peripheral CLL cells.^32^

But what is the significance and impact of higher CD86 expression on the surface of CLL cells? T cells in CLL show several phenotypic and functional abnormalities, resulting in an ineffective anti‐tumor immune response. The T cells are typically pseudo‐exhausted, showing an altered cytokine profile, reduced proliferation and cytotoxic function, and increased expression of inhibitory molecules (CTLA‐4, PD‐1, TIM‐3).^33^, ^34^ Reduced CD28 expression compared to healthy individuals has also been reported.^34^ The exhausted T‐cell phenotype was one of the major contributors to the CAR‐T‐cell treatment failure in CLL.^35^ In addition, CLL is characterized by an increased number of Tregs, which also have an enhanced suppressive capacity compared to normal Tregs.^36^, ^37^ Since CD86 can activate not only effector T cells but also Treg cells, it is possible that in CLL, the tumor cells suppress the T‐cell immune response via the CD86‐Treg connection, and the anti‐tumor response capability of the immune system is reduced. This hypothesis is supported by studies showing that CLL patients with higher CD86 expression required treatment earlier than CD86‐negative patients.^30^, ^38^

**FIGURE 2:**
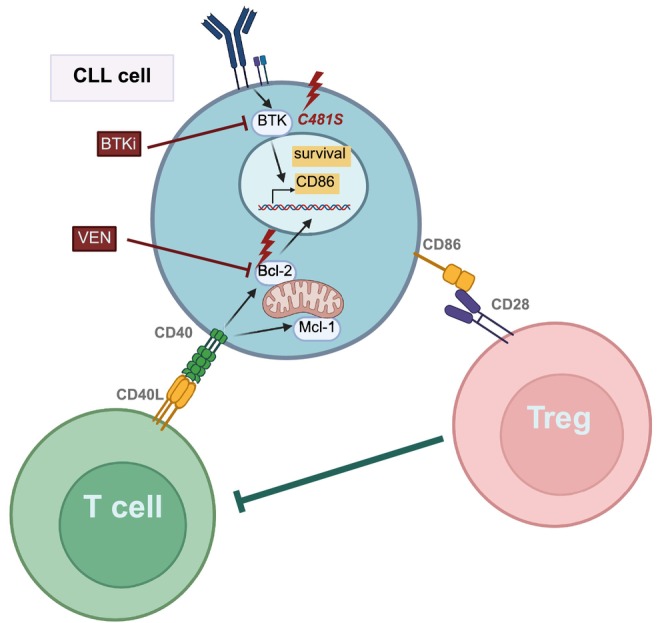
The role of CD86 in the survival of CLL cells. CD86 expression is lower on CLL cells than healthy B cells; however, several signals can increase its expression, such as BTK activation and CD40 stimulation. The Bcl‐2 inhibitor venetoclax (Ven) and BTK inhibitors (BTKi) decrease CD86 expression, but at the onset of resistance, it is increased. Therefore, CD86 is a useful indicator of the activity of these signaling pathways. Furthermore, CD86 has been observed to facilitate the activation of Treg cells, which exert an immunosuppressive effect that contributes to the survival of tumor cells. BTK, Bruton's tyrosine kinase; Mcl‐1, Myeloid cell leukemia 1; Treg, regulatory T cell.

Although CD86 expression influenced the necessity of treatment, different treatments also affected the expression of this protein. Lenalidomide, an immunomodulatory drug, is thought to repair the immune synapse by increasing the expression of costimulatory molecules, including CD86, in both in vitro cultures^39^ and in patient samples.^40^ The oligodeoxynucleotide CpG‐685, an immunomodulatory agent in CLL, which binds to toll‐like receptors (TLRs), also increased CD86 levels.^41^ In contrast, the reversible BTK inhibitor ARQ531 reduced CD86 expression, as did the irreversible BTK inhibitor ibrutinib.^42^ These results are not surprising, as previous studies showed that BCR activation or BTK activity increased CD86 expression, thus their inhibition reduced CD86 levels (Figure 2.). Selicrelumab, a CD40 antagonist, also increased the CD86 expression by activating the CD40 signaling pathway and CLL cells.^43^ In these experiments, the stimulation of CD40 sensitized CLL cells to anti‐CD20 therapy and increased the expression of anti‐apoptotic members of the Bcl‐2 family proteins (e.g., Bcl‐xl, Mcl‐1),^43^ rendering CLL cells resistant to conventional chemotherapy^44^ or therapy with the Bcl‐2 inhibitor venetoclax^45^ (Figure 2.). These results may be related to increased CD86 expression observed in resistant CLL cells: in a patient treated with venetoclax, the CD86 levels decreased during the treatment and then increased again a few months before the onset of clinical resistance.^46^ In vitro experiments also uncovered that the expression of Ki67, PD‐1, and CD86 was increased in venetoclax‐resistant CLL cells.^47^ Similar phenomena were detected in ibrutinib‐treated patients: comparing untreated, ibrutinib‐treated, and ibrutinib‐resistant cases, resistant cases had significantly higher CD86 expression than cases in other groups. Ibrutinib‐resistant cases with high CD86 expression harbored BTK^C481S^ mutation. Similarly to venetoclax treatment, in the same patient, the CD86 decreased at the beginning of the ibrutinib therapy and later increased before the onset of ibrutinib resistance.^48^

Ibrutinib inhibited BCR and NF‐kB signaling pathways by reducing phospholipase C gamma 2 (PLCγ2) phosphorylation and the expression of NF‐kB p50 protein.^49^ Herman et al. also detected decreased CD86 and CD69 expressions during ibrutinib treatment. Since the activity of the former pathways influences CD86 expression, increased CD86 expression in ibrutinib‐resistant cases indicates the recovery of the BCR pathway; thus, it can be a suitable marker for monitoring ibrutinib resistance in CLL cells. Besides CLL cells, ibrutinib treatment affected other immune cells (e.g., T cells, NK cells, monocytes) involved in the anti‐tumor effect, restoring them to normal levels. The number and the proportion of Tregs decreased, and the effector functions of CD8‐positive T cells were restored.^50^ The following question is posed: were these changes due to the reduced number of CLL cells or the T‐cell‐specific inhibitory effect of ibrutinib on IL2‐inducible T‐cell kinase (ITK).^51^ A subsequent study answered this question by investigating the impact of the second‐generation, more specific BTK inhibitor, zanubritinib, on T cells and found similar results: reduction in the number of Treg cells and the restoration of T‐cell effector functions.^52^ Therefore, it was more likely that the T cells were altered by CLL cells, and CD86 may play a role in this process.

### 6.2 CD86 expression on B‐cell acute lymphoblastic leukemia cells

**FIGURE 3:**
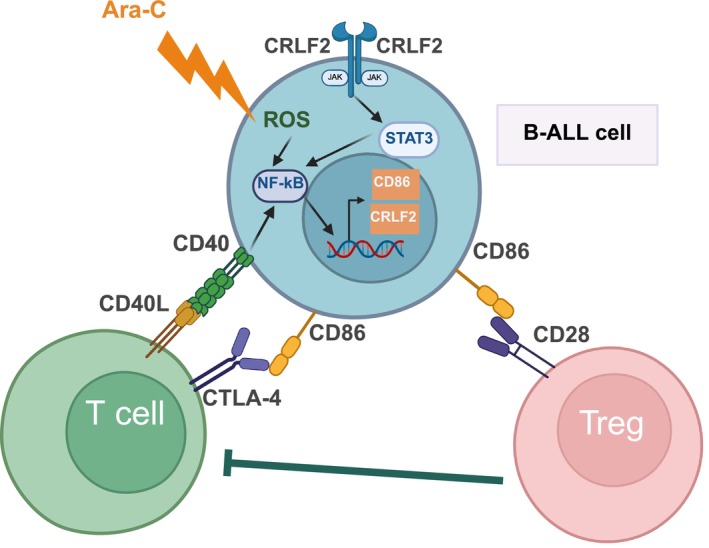
CD86 expression of acute B‐cell lymphoblastic leukemia cells. In half of the acute B‐cell lymphoblastic leukemia cases, blasts express CD86 on their surface. In cases with CRLF2 overexpression, the constitutive JAK–STAT signaling pathway and, in other cases, the active CD40 pathway can induce CD86 expression on leukemia cells. Furthermore, it has been demonstrated that specific treatments, such as Ara‐C, can also induce CD86 expression via NF‐kB activation. CD86 may inhibit the activation of the effector T cells by CTLA‐4 binding or the activation of Tregs via CD28. Ara‐C, cytosine arabinoside; CRLF2, cytokine receptor‐like factor 2; ROS, reactive oxygen species.

In B‐cell acute lymphoblastic leukemia (B‐ALL), leukemic cells are similar in their phenotype to normal, progenitor cells, hematogones. Genome‐wide analysis of 270 B‐ALL cases and flow cytometry‐validated protein analysis of 135 B‐ALL cases revealed that half of them had higher CD86 expression than normal progenitor cells.^53^ Similar results were obtained in another study of 90 pediatric B‐ALL cases, in which 56.7% of the cases had aberrant, higher CD86 expression on leukemic blasts compared to progenitor cells. Analysis of normal hematogones revealed that neither the early nor the late progenitors express CD86. Treatments are known to affect antigen expression, as steroid treatment promotes the maturation of blasts, resulting in a decline in CD10 expression and an increase in CD20 expression.^54^, ^55^ CD86 expression in MRD‐positive cases proved to be a useful MRD marker because it showed stable expression: in the majority of childhood B‐ALL cases (40/56, 71%), the CD86 expression was unchanged or increased on day 15 of treatment compared to diagnosis.^56^ Because these treatments mainly consisted of steroids, we can state that the CD86 expression is not affected by steroid treatment. A later component of the leukemia treatment, cytosine arabinoside (Ara‐C), which stops the cell cycle and tumor cell division, more than doubled the CD86 levels in 50% of cases.^57^ Fan D. et al. showed that Ara‐C treatment activated the NF‐kB pathway by reactive oxygen species.^58^, ^59^ Probably, NF‐kB pathway activation induces CD86 expression in these cells. In addition, similarly to CLL cells, CD40‐CD40L binding increased CD86 expression in ALL cells^60^ (Figure 3.).

Analyzing surface markers and genetic subtypes, Coustan‐Smith et al. found that in the hyperploid ALL subgroup, the CD86 expression was higher than in other groups, while subsequent studies found no correlation between CD86 expression and genetic subtypes in leukemic cells.^56^, ^61^ Another study investigated the abnormal expression of cytokine receptor‐like factor 2 (CRLF2) and established that cases exhibiting overexpressed CRLF2 had a higher proportion of CD86‐positive cases than cases demonstrating low/negative CRLF2 expression.^62^ Furthermore, CD86‐positive cases had higher CRLF2 mRNA levels. Overexpression of CRLF2 is frequent in Ph‐like ALL cases and activates JAK2‐mediated STAT3 and STAT5 pathways, triggering cell proliferation. In some cancers (e.g., DU145 prostate cancer, A2058 melanoma cell line) and mouse DC cells, STAT3 was found to induce a constitutive basic phosphorylation of RelA, an NF‐kB protein, which could increase CD86 expression.^63^ Similar activation may occur in leukemic cells, and CD86 expression is a sign of this activation (Figure 3.). Furthermore, B‐ALL was also characterized by immune evasion through the modification of the microenvironment and the function of its cells by leukemia cells: the differentiation of monocytes^64^ and the alteration of T‐cell function, as evidenced by the anergy of CD8‐positive T cells^65^ were found. Additionally, an increase in the Tregs ratio and the immune suppressive activity was observed in leukemic patients.^66^, ^67^ Thus, CD86 expression on the surface of leukemic blasts may play a role in the inactivation of effector T cells by CTLA‐4 binding or the activation of Tregs via CD28.

### 6.3 CD86 in multiple myeloma (MM)

MM is characterized by the proliferation of clonal plasma cells in the bone marrow. On normal plasma cells and plasmablasts, the expression of CD86 was higher than on naive B cells,^68^ which is not surprising since its level increases on activation. The CD86 expression on MM cells was investigated in numerous studies, and found that approximately 50% of MM samples or cell lines were positive for CD86 at protein or RNA levels.^69^, ^70^, ^71^ Furthermore, abnormal plasma cells in newly diagnosed MM cases had a lower level of CD86 expression than the normal plasma cells.

Investigating the prognostic impact of CD86 expression, Brown RD et al. found that CD86‐positive MM cases had shorter survival and greater tumor mass than CD86‐negative cases.^72^ In their studies, different populations of MM cells were distinguished by their CD45 expression: primitive (CD38^hi^CD45^hi^) and immature (CD38^hi^CD45^lo^) myeloma cells had higher CD86 levels than mature (CD38^hi^CD45^neg^) myeloma cells, while patients with mature myeloma cells had poorer prognoses.^69^, ^72^ Moreover, their in vitro study on isolated myeloma cells showed that adding CD40L did not increase CD86 protein levels.^72^

Kinoshita R et al. investigated whether the level of soluble CD86 beside surface CD86 can be a prognostic factor. A study of 103 patients revealed that those with high levels of sCD86 exhibited a more severe disease and diminished overall survival in contrast to those with lower sCD86 levels. In these patients, serum sCD86 levels showed a weak correlation with surface CD86 expression.^7^

Another workgroup used the CoMMpass and the molecular database from the University of Arkansas to investigate the cytogenetic and molecular subgrouping of CD86‐positive MM cases.^73^ According to the CoMMpass database, high‐risk MM cases with t(14;16) or t(14;20) had higher CD86 expression. Based on molecular subgroups, cases belonging to the MF group, in which the MAF gene and its associated signaling pathway were mutant, showed higher CD86 expression. In this subgroup, cases with t(14;16) or t(14;20) could also be found. These results suggest that cases with high CD86 expression likely have a poor prognosis.

**FIGURE 4:**
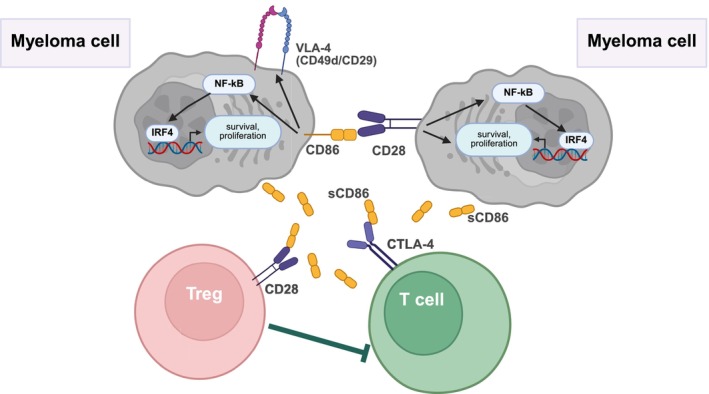
The significance of CD86 expression in the survival of myeloma cells. In a significant proportion of cases of multiple myeloma, the expression of CD86, along with CD28, has been observed on the surface of malignant plasma cells. They enhance myeloma cell survival via activation of the NF‐kB signaling pathway and play a role in increasing the expression of surface integrin (e.g., ITGβ1‐CD29). Moreover, the impact of these molecules extends to the myeloma microenvironment, where they regulate T cells through the release of soluble CD86 (sCD86), reducing their anti‐tumor and increasing their tumor‐promoting effects. VLA‐4, very late activation antigen 4; IRF4, interferon regulatory factor 4; sCD86, soluble isoform of CD86.

Gavile et al. investigated not only the CD86 but also the CD28 expression of myeloma cells. Surprisingly, they found that CD86 is often co‐expressed with CD28 on myeloma cells. This is interesting because CD86 is a ligand of CD28, and CD28 is important for the survival of long‐lived plasma cells.^74^ Myeloma cells expressing CD86 and CD28 can likely activate each other, promoting their survival and reducing their dependence on the microenvironment (Figure 4.). Silencing either molecule supported this theory by increasing the death of myeloma cells. Cell death induced by inhibiting or knocking down CD86 was partially caspase‐dependent, as the general caspase inhibitor could only partially decrease the proportion of apoptotic cells.

Overexpression of CD86 decreased the CD28 expression of MM cells, while silencing CD28 increased the level of CD86. Silencing CD86 or CD28 also reduced the level of IRF4, which is required for the survival of MM cells.^75^ In these cases, the decrease in IRF4 level was probably caused by a decreased NF‐kB activity induced by CD86 or CD28, as both molecules can activate this signaling pathway in MM cells,^76^ an important regulator of IRF4 expression^77^ (Figure 4.).

In addition, mutation experiments of the intracellular part of the human CD86 showed that its cytoplasmic domain was required for the surface expression of integrins (e.g., ITGß1 [CD29] and ITGß7), which are crucial in the interaction of MM cells with their microenvironment (Figure 4.). Furthermore, the complete CD86 molecule was also required for MM cells' survival when they were treated with anti‐Bcl‐2/xl and dexamethasone, but not with a proteasome inhibitor.^73^ Presumably, survival signals activated by CD86 (e.g., NF‐kB signaling pathway) or by binding to stromal cells could promote the survival of MM cells by blocking the inhibitory effect of Bcl‐2. In contrast, these pathways were unlikely to play a role in survival with proteasome inhibitors. These results suggest that CD86 is involved in the survival of MM cells and stromal cell binding.

### 6.4 CD86 in other hematological malignancies

CD86 is also expressed on other B‐cell lymphoma cells. In splenic marginal zone lymphoma (SMZL) the level of CD86 increased following the stimulation of their toll‐like receptors. In these cells, toll‐like receptors (e.g., TLR1/2, TLR2/6, and TLR9) activated the MAPK, IRAK, and NF‐kB signaling pathways, which could increase cell proliferation and elevate the level of CD86.^78^ In primary effusion lymphoma (PEL) cells, pomalidomide treatment decreased IRF4, c‐Myc, and CK1a levels and increased the expression of ICAM‐1 and CD86.^79^ These molecules activated T‐cell and NK‐cell toxicity and thus contributed to increased lymphoma cell death. In this case, the presence of CD86 facilitated the anti‐tumor effect.

Besides ALL cases, the expression of CD86 was also detected in AML cases. CD86 positivity was more frequent in AML cases belonging to the M4 and M5 subgroups of the French‐American‐British (FAB) classification. In 110 cases of de novo AML, cases with CD86‐positive blasts (54%), regardless of CD34 and CD14 expression, could differentiate into monocytic/dendritic cells. Thus, CD86 could be a useful marker for monocytic/dendritic cell AML.^80^

Anti‐leukemic treatments such as radiation or Ara‐C can influence the expression of costimulatory molecules on the surface of leukemic cells. Ara‐C treatment of AML cells increased the expression of CD80 and CD86 in both in vitro and in vivo experiments, and it increased T‐cell‐induced cell lysis.^81^ These results are in contrast to database analysis, which showed that AML cases with higher CD86 expression had poorer prognoses.^82^ Moreover, the number of inflammatory immune cells (CD8‐positive T cells, dendritic cells, macrophages, NK cells, and Th1 cells), which can support tumor growth, was higher in the CD86‐high group. The response to immune checkpoint inhibitors also negatively correlated with CD86 expression.^82^ This finding indicates that CD86 facilitated the activation and approach of immune cells to tumor cells, suggesting its potential tumor‐promoting role in these processes.

## 7 SUMMARY

From the above, we can conclude that CD86 is expressed on the surface of many tumor cells and is often associated with poor prognosis or drug resistance. CD86 can indeed activate T cells, but it may promote the immunosuppressive effect of Treg cells, or its constitutive, high expression can inhibit T‐cell function or CAR‐T‐cell therapy via CTLA‐4. The exact mechanism remains to be elucidated, but it has been hypothesized that high CD86 expression indicates the activation of a signaling pathway mediated by NF‐kB or the B‐cell receptor, which is associated with a worse prognosis in B‐cell malignancies.

In contrast, certain treatments can elevate CD86 levels, which promote the anti‐tumor response via activating effector T cells.

Thus, as a real double agent, the role of CD86 is not clear in different hematological tumors, but its expression and function should be considered in the applied treatments.

## AUTHOR CONTRIBUTIONS

**Gábor Barna:** Conceptualization; writing – original draft; visualization. **Gábor Szalóki:** Writing – review and editing. **Ágnes Márk:** Writing – review and editing. **Anna Hunyadi:** Writing – review and editing. **Csilla Kriston:** Conceptualization; writing – review and editing; visualization.

## CONFLICT OF INTEREST STATEMENT

The authors declare no conflict of interest.
